# Temperature Influence on PI/Si_3_N_4_ Nanocomposite Dielectric Properties: A Multiscale Approach

**DOI:** 10.3390/polym13121936

**Published:** 2021-06-10

**Authors:** Mohammed Houssat, Christina Villeneuve-Faure, Nadine Lahoud Dignat, Marie-Laure Locatelli, Jean-Pascal Cambronne

**Affiliations:** LAPLACE, Université de Toulouse, CNRS, INPT, UPS, 31062 Toulouse, France; houssat@laplace.univ-tlse.fr (M.H.); marie-laure.locatelli@laplace.univ-tlse.fr (M.-L.L.); jean-pascal.cambronne@laplace.univ-tlse.fr (J.-P.C.)

**Keywords:** polymer nanocomposite, interphase, multi-scale characterization, dielectric permittivity, breakdown strength, EFM, PF-QNM

## Abstract

The interphase area appears to have a great impact on nanocomposite (NC) dielectric properties. However, the underlying mechanisms are still poorly understood, mainly because the interphase properties remain unknown. This is even more true if the temperature increases. In this study, a multiscale characterization of polyimide/silicon nitride (PI/Si_3_N_4_) NC dielectric properties is performed at various temperatures. Using a nanomechanical characterization approach, the interphase width was estimated to be 30 ± 2 nm and 42 ± 3 nm for untreated and silane-treated nanoparticles, respectively. At room temperature, the interphase dielectric permittivity is lower than that of the matrix. It increases with the temperature, and at 150 °C, the interphase and matrix permittivities reach the same value. At the macroscale, an improvement of the dielectric breakdown is observed at high temperature (by a factor of 2 at 300 °C) for NC compared to neat PI. The comparison between nano- and macro-scale measurements leads to the understanding of a strong correlation between interphase properties and NC ones. Indeed, the NC macroscopic dielectric permittivity is well reproduced from nanoscale permittivity results using mixing laws. Finally, a strong correlation between the interphase dielectric permittivity and NC breakdown strength is observed.

## 1. Introduction

Polymer dielectric materials are commonly used in advanced electronic devices and electric power systems’ insulation. However, even if polymer dielectrics possess excellent mechanical properties and high electric breakdown strength, their dielectric properties (leakage current, space charge accumulation, breakdown strength, etc.) at high temperature need to be improved [1,2]. Particularly, for polyimide (PI), which is broadly used in the fields of electronic packaging and automotive applications, a degradation of its electrical resistivity, dielectric properties, and/or failure field [3,4,5] is observed at high temperature. According to the literature, polymer-nanocomposites (NCs) appear as the best candidates to improve the polymer dielectric properties at both ambient [6,7] and high temperature [1,8]. PI-based NCs, with a small amount of nanofillers, exhibit improved mechanical, thermal [9,10,11], and dielectric properties at high temperature, as the limitation of space charge accumulation [12,13], the improvement of breakdown strength [12,14,15] and/or energy density [3,15,16] compared to the neat PI. To explain the influence of nanofillers on NC properties, T.J. Lewis proposed the hypothesis, in 1994, that a transition area named the “interphase” is created between the nanofillers and the polymeric matrix [17]. Since then, different models have been proposed to describe the interphase morphology and structure [17,18,19,20]. In addition to these theoretical approaches, some authors have proposed to extract the interphase properties from macroscale experimental characterization methods [21,22,23,24]. However, the interphase properties remain challenging to characterize and their impact on NC ones are, consequently, poorly understood. To characterize interphase properties at the local scale, techniques derived from the Atomic Force Microscopy (AFM) were used: the Peak Force Quantitative NanoMechanical (PF-QNM) mode for the interphase mechanical properties and dimension determination [25,26], the Electrostatic Force Microscopy (EFM) mode for the dielectric permittivity [27,28,29], and the Kelvin Probe Force Microscopy (KPFM) for space charge measurements [30,31]. All of these existing studies only provide an interphase characterization at room temperature and a partial description of dielectric properties as some assumptions are requested for their entire determination. For example, an attempt was made to determine the interphase width and dielectric permittivity from the same EFM phase shift measurement for epoxy/BaTiO_3_ NC [29]. However, the limits in spatial resolution of the EFM were not considered even when the bump height and the lift were obviously high.

In this context, the originality of our approach is to combine PF-QNM and EFM measurements to probe, respectively, the interphase width and dielectric permittivity at various temperatures. These nanoscale properties are then correlated to macroscale NC dielectric permittivity and breakdown strength to provide a new insight into the impact of interphase on NC properties. Because it has improved properties (e.g., dielectric breakdown) at high temperature [12,32], PI/silicon nitride (Si_3_N_4_) NC is investigated in this study. Moreover, a nanoparticle (NP) surface treatment will be used to functionalize Si_3_N_4_ NP and consequently modify the interphase dimension and/or properties [6,33,34,35].

## 2. Materials and Methods

### 2.1. Materials Processing

In this paper, a PI-based NC is investigated using a 1 wt.% addition of Si_3_N_4_ spherical amorphous NPs provided by SkySpring Nanomaterials Inc. (Houston, TX, USA). The PI host matrix is processed using a mix of commercial biphenyltetracarboxylic dianhydride and p–phenylene diamine precursor monomers (BPDA-PDA) supplied as a polyamic acid (PAA) precursor solution in N-methyl-2-pyrrolidone solvent. Raw materials properties are reported in Table 1. The NP surface functionalization is obtained by using (3-aminopropyl) triethoxysilane (APTES, 98%) purchased from Sigma-Aldrich (St Quentin Fallavier, France).

**Figure 1:**
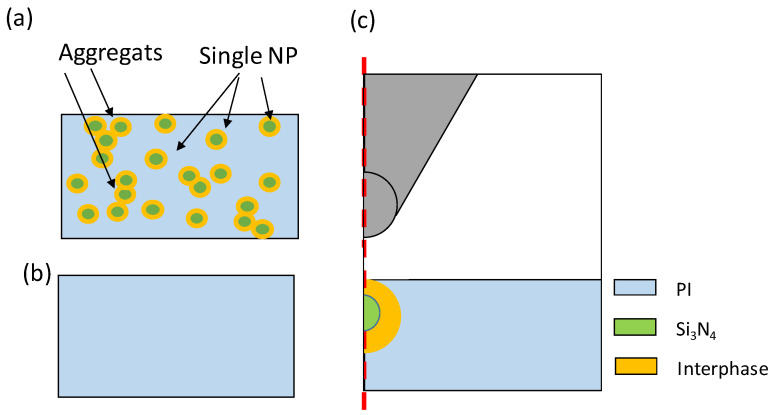
Graphical representation of the: (**a**) nanocomposite and (**b**) bare polyimide (PI) layers. (**c**) Scheme of the 2D-axisymetric FEM model developed for static dielectric permittivity determination from EFM measurements.

The NC fabrication process is composed of four steps (detailed in [25]). (i) 5 g of PAA are mixed for 15 min, with 0.05 g of Si_3_N_4_-NP (treated or not with silane coupling agent) to obtain a homogeneous 1 wt.% aqueous solution. (ii) One hour of ultra-sonication process (70 °C, 300 W) and 25 min centrifugal decantation (14,400 rpm) are used to reduce the size of agglomerates formed during the mechanical dispersion. After that, only the supernatant mixture is used (1 mL). (iii) The NC solution is spin-coated (30 s at 3000 rpm), on 2-inch highly-doped Si wafers (N-type, resistivity: 0.002–0.008 Ω.cm, thickness: 275 ± 25 μm) to obtain a thin film with controlled thickness. (iv) The obtained PAA/Si_3_N_4_ NC thin films are annealed in air at 100 °C for 1 min and at 175 °C for 3 min. Finally, in order to obtain the PI imidization from PAA, the thin films are thermally cured in a SPX Blue-M convection oven in nitrogen atmosphere. Pure PI films have been elaborated as well for comparison (using the same two last process steps). The graphical representation of both neat PI and NC layers is depicted in Figure 1a,b, respectively. Concerning the NC, the material morphology presents NP homogeneously dispersed in the volume and aggregates [25,36].

NC films with thicknesses of 2.3 and 3.4 µm for untreated and treated NP, respectively, were estimated using a KLA-Tencor mechanical profilometer. For pure PI film, the thickness was 5.8 µm.

For macroscale characterization (dielectric spectroscopy and breakdown strength), capacitive metal–insulator–semiconductor (MIS) structures are needed. To produce such structures a 150 nm-thick gold layer is deposited by sputtering over the entire top surface of the film samples. A wet etching step through a resin mask, made by photolithography, permitted for definition of the top circular electrode with a diameter of 5 and 0.6 mm for dielectric spectroscopy and breakdown measurements, respectively. Moreover, as PI is sensitive to the water absorption, a heating treatment (150 °C for 48 h) is applied to PI and NC films before macroscopic measurements.

### 2.2. Macroscopic Dielectric Properties Characterization

The dielectric properties were measured by broadband dielectric spectroscopy (DS) using a Novocontrol Alpha-A spectrometer. Measurements were performed under a nitrogen gas flow, in a temperature range from −150 to 350 °C (with steps of 10 °C) and in a frequency range from 0.1 Hz to 100 kHz. The data were obtained in the form of the dielectric complex permittivity as shown in Equation (1):

(1) $$\varepsilon^{*}=\varepsilon^{\prime }-i\;\varepsilon^{\prime \prime },\;$$

where *ε*′ and *ε*″ are, respectively, the dielectric permittivity and the dielectric loss index.

The breakdown field measurements were carried out using a Signatone S-1160 probe station equipped with micrometric positioners and a sample holder regulated at 25, 100, 200, and 300 °C thanks to the S-1600R heating system. A DC voltage ramp of 150 V/s was supplied using a FI 9035HT source. When the breakdown occurs, the used voltage source switches into a current source applying a short-circuit limited to 1 mA. The breakdown electric field *F_br_* has been calculated using Equation (2):

(2) $$F_{br}=\frac{V_{br}}{d},\;$$

where *V_br_* is the measured breakdown voltage and *d* the insulating film thickness.

A Weibull statistical analysis on a population of 10 samples was performed to extract the scale parameter of the breakdown for each temperature.

### 2.3. Nanoscale Characterization

AFM measurements were performed using a Bruker Multimode 8 apparatus. PF-QNM mode was used to map the surface topography and the mechanical properties of NC films using a TAP525 tip. A contact force of 600 nN was applied to obtain a 2 nm-deformation on the bare PI. According to the stiff contact and the low adhesion observed on PI, the Young’s modulus is calculated using the Derjaguin–Muller–Toropov (DMT) model [37,38]. To insure reliable and quantitative results a three-step calibration process was applied [36]: (i) Force–distance curves were used to determine static and dynamic deflection sensitivity on hard sapphire sample. (ii) Scanning electron microscopy was used to determine tip geometrical parameters in order to calculate the tip spring constant *k* using Sader method. (iii) The effective tip radius was determined using a polystyrene (2.7 GPa modulus) reference film whose mechanical properties are close to PI ones. The measurements were done using 384 × 384 pixels on a 2 µm × 2 µm scanned area, which corresponds to a pixel size of 5.2 nm. EFM and PF-QNM measurements were performed on an isolated NP protruding over the surface (Figure 1a). Indeed, around aggregates the interphase can be defined unambiguously.

EFM mode was used to probe the dielectric permittivity of NC films at the nanoscale [27,39]. A PtIR-coated silicon tip with a resonance frequency$\;f_{0}$ of 66.1 kHz, a spring constant *k* of 2.74 N/m and a curvature radius *R_C_* of 26 nm was used. The tip frequency shift Δ*f*_0_(*V*_0_) was probed for DC voltage *V*_0_ of 0 or 10 V on the tip with a 50-nm lift.

The resulting frequency shift parameter *a_Δf_* was determined using Equation (3) [39]:

(3) $$a_{\Delta f}=\frac{\Delta f_{0}\left(10\;V\right)-\Delta f_{0}\left(0\;V\right)}{{\left(10\right)}^{2}},$$

where Δ*f*_0_ (10 V) and Δ*f*_0_ (0 V) are the frequency shifts measured with a DC voltage of 10 V and 0 V applied on the tip, respectively.

To extract the static relative dielectric permittivity from this measurement a 2D-axisymetric Finite Element Model (FEM) [36,39,40] was developed and solved on COMSOL. The model accounts for the NP (radius 10 nm and relative dielectric permittivity $\varepsilon_{NP}=7.5$), the PI matrix (NC films thickness and relative dielectric permittivity $\varepsilon_{m}$), the interphase (thickness *W_i_* and relative dielectric permittivity $\varepsilon_{i}$ to be determined), and the surrounding atmosphere (air box whose dimensions are fixed to avoid edge effect). The AFM tip, located at 50 nm from the surface was represented as a 10 µm height truncated cone (14° of aperture angle), ending with a semi-spherical apex (*R_C_* = 26 nm). The model scheme was summarized in Figure 1c. The dielectric layer backside was grounded whereas a potential *V*_0_ was applied to the AFM tip. Mesh was refined and optimized close to the tip radius and in the NC layers to improve calculation accuracy.

To extract the relative dielectric permittivity, a four-step method was needed. First, the Poisson’s equation, without charge density, was solved in air and in the dielectric film to determine the electric field $\vec{E}$ distribution as following:

(4) $$\vec{\nabla }\left(\varepsilon_{0}\varepsilon_{r}\vec{E}\right)=0,\;$$

where $\varepsilon_{0}$ is the vacuum dielectric permittivity and $\varepsilon_{r}$ is the relative dielectric permittivity.

Secondly, the electrostatic force *F_e_* acting on the probe was deduced as:

(5) $$F_{e}=\iint_{tip}^{\;}\frac{\varepsilon_{0}}{2}{||E||}^{2}n.dS,\;$$

Third, the capacitance second derivative *d*^2^*C*/*dz*^2^ was deduced from the electrostatic force *F_e_* gradient, as:

(6) $$\frac{d^{2}C}{dz^{2}}=2\frac{dF_{e}}{dz},\;$$

Finally, the frequency shift parameter *a*_Δ*f*_ was computed as follows [39]:

(7) $$a_{\Delta f}=\frac{f_{0}}{4k}\frac{d^{2}C}{{dz}^{2}},\;\;$$

where *f*_0_ is the resonance frequency, *C* is the capacitance between the AFM tip and the sample, *z* is the vertical distance, and *k* is the stiffness of the cantilever.

Results emphasize that the computed frequency shift parameter was negative and increased when the material permittivity decreased.

## 3. Results and Discussion

### 3.1. Dielectric Properties of NCs at Macroscale

**Figure 2:**
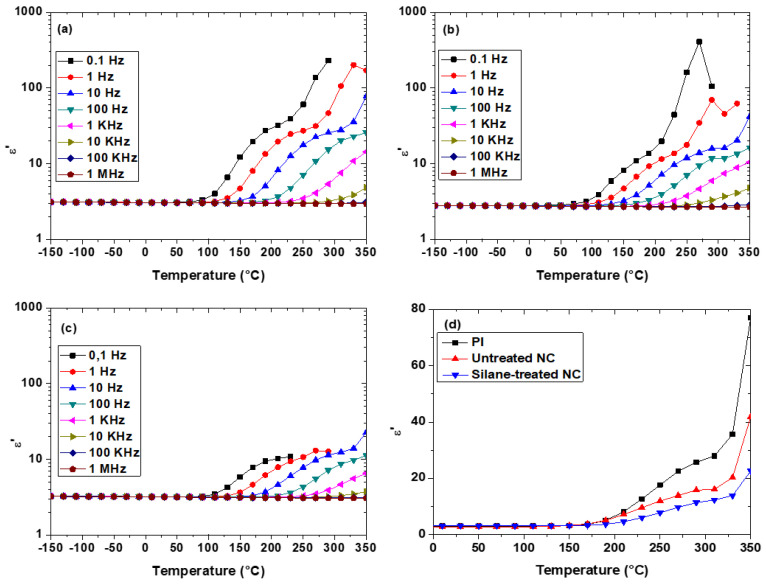
Temperature dependence of the real part of the relative dielectric permittivity for (**a**) neat PI, NC with (**b**) untreated and (**c**) treated NPs. (**d**) Evolution of the relative dielectric permittivity at 10 Hz as a function of temperature.

For the PI sample, an increase of the relative dielectric permittivity is observed at temperatures higher than 100 °C for different frequencies (Figure 2a). This relaxation phenomenon is usually attributed to the electrode’s polarization due to space charge accumulation [41]. Indeed, when thermally activated, positive (or negative) charge carriers can drift to the negative (or positive) polarized electrode in order to build up a space charge at the sample-electrode interface. This is related to the electrical conduction in PI films which is influenced by macroscopic migration of H^+^ ions (coming from unreacted PAA [42]) to the interfaces. Figure 2b,c shows the same relaxation phenomenon (relative dielectric permittivity increased) at high temperature for PI/Si_3_N_4_ NC with untreated and treated NP, respectively. So, quite the same physical mechanism seems to be involved in PI and PI/Si_3_N_4_ NC concerning the dielectric permittivity.

Figure 2d compares PI and NC dielectric permittivities at low frequency (i.e., 10 Hz). At low temperature (T < 150 °C), PI and NC show similar and almost constant dielectric permittivities. This behavior was already observed for 1 wt.% PI/Si_3_N_4_ [12], PI/BaTiO_3_ [16], and PI/PLZT [43] NC. At high temperature (T > 150 °C), the increase of dielectric permittivity with temperature increase is faster for the PI compared to both NC. Moreover, the dielectric permittivity for NC with treated NP remains the lowest whatever the temperature is. This difference in behavior could be related to phenomena occurring within the interphase and to its property’s evolution with temperature. The most likely explanation is that, at high temperature, where ion charge carriers are highly mobile, the presence of NP seems to disturb the ions transport. Indeed, the interphase region around NP could be a region where H^+^ ions are blocked, limiting their transport to the sample–electrode interface. Moreover, the interphase and the matrix permittivities could change with the temperature and modify the electric field distribution. In all of the cases, the silane coupling agent, acting as an anchoring link for the PI macromolecular chains on the Si_3_N_4_ particles, modifies the interphase dimension and properties, which implies a more important modification of the macroscale properties.

**Figure 3:**
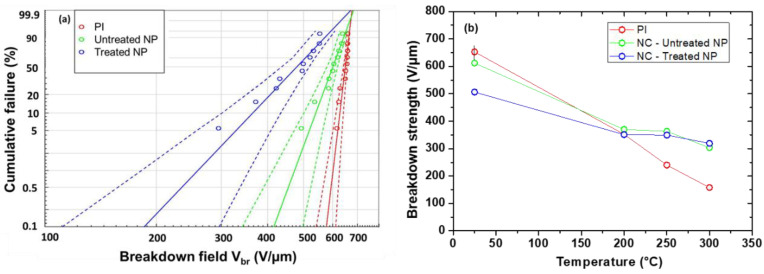
(**a**) Weibull statistics for breakdown results of neat PI, PI/Si_3_N_4_ and PI/Si_3_N_4_-silane samples at 25 °C (dashed lines correspond to 90% confidence intervals). (**b**) Temperature dependence of breakdown strength for neat PI, PI/Si_3_N_4_, and PI/Si_3_N_4_-silane samples.

Breakdown tests are performed on neat PI and PI/Si_3_N_4_ NC with untreated and silane-treated NP. Figure 3a shows breakdown results under 25 °C for all samples. A Weibull statistical analysis is performed giving a breakdown strength *F_br_* value at each temperature for all samples and plotted in Figure 3b. At low temperature (<200 °C), PI presents a slightly higher breakdown strength than both NC. At room temperature, this behavior was already observed for 1 wt.% PI/Si_3_N_4_ [12] and PI/BaTiO_3_ [16] NC. However, at high temperature (> 200 °C), PI presents a much lower breakdown strength compared to NC ones. In comparison with neat PI, which becomes a semi-insulating material above 200 °C, it seems that PI/Si_3_N_4_ NC with treated or untreated particles keep good insulating behavior up to 300 °C. Indeed, at 300 °C the breakdown strength is improved by a factor of 2 compared to PI. A similar phenomenon was observed for PI/BN NC [32]. This phenomenon could be related to the “Thermal stabilization effect” hypothesis introduced by Yang et al. [35] for PI/Al_2_O_3_ NC. According to this effect, the interphase region within NC contains new traps compared to the neat matrix, which modifies the charge trapping and transport mainly at high temperature. Hence, the charge injection and accumulation are restrained, the local electric field is alleviated, and the breakdown field is increased.

From a macroscale point of view, the presence of NP (treated or not) decreases the relative dielectric permittivity (Figure 2d) and improves the breakdown strength (Figure 3b) at high temperature (T > 150 °C). The same phenomenon was observed for other PI-based NCs [12,32]. According to the literature, this improvement is related to the interphase properties [33] but the underlying physical phenomena is not completely understood. So, in order to identify this behavior, the interphase properties at high temperature need to be characterized at the local scale. To reach this goal PF-QNM and EFM were used, in the following section, to determine the interphase dimension and relative dielectric permittivity.

### 3.2. Interphase Properties at Nanoscale

**Figure 4:**
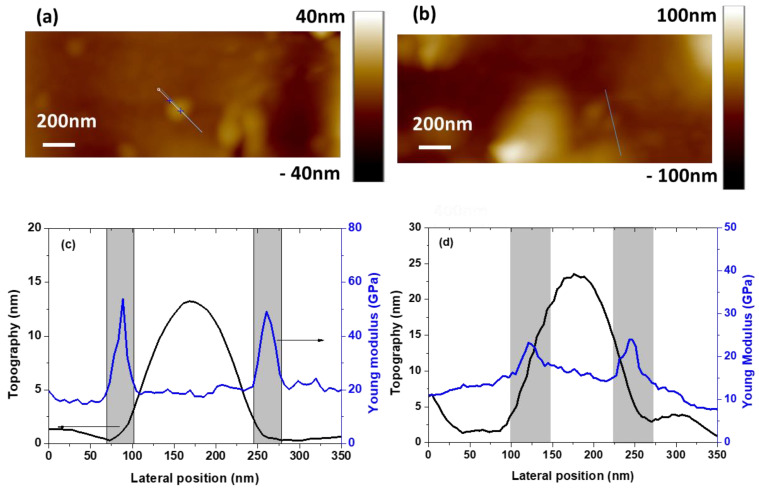
PF-QNM topography map for nanocomposite with (**a**) untreated and (**b**) treated NPs. Comparison of topography and Young’s modulus profiles over protruding NPs (pointed by straight line on topography map) for (**c**) untreated and (**d**) treated NPs.

The NC surface topography comparison between untreated (Figure 4a) and treated (Figure 4b) NP emphasizes that protruding isolated NP could be identified. For untreated NP, Figure 4c compares surface topography and Young’s modulus profiles over an isolated NP. On the topography profile, a 15-nm height bump reveals the presence of the NP. On the Young’s modulus profile, two areas are identified. The first one (white area), located above and laterally far from the NP, has a Young’s modulus of 20 GPa, and is attributed to the matrix. The second one (grey area), located around the NP, exhibits an apparent Young’s modulus higher than that of the matrix. This area is attributed to the interphase and its width *W_i_* is determined as the lateral dimension of this area. The interphase width is determined for at least ten NPs and a mean value of 30 ± 2 nm is obtained. For treated NPs, similar results are obtained for both topography and Young’s modulus (Figure 4d). A similar bump is chosen (i.e., height close to 15 nm) and as previously, the interphase width is determined for at least ten NPs and a mean value of 42 ± 3 nm is obtained. The interphase is thicker for treated NPs than untreated ones. This result confirms that the coupling agent used for the NP surface treatment increases the interaction strength between the NPs and the matrix, which leads to the interphase dimension increase [33,34,35].

Whatever the NP treatment, the Young’s modulus of the interphase is higher than in the matrix. This phenomenon was already observed for various NCs as epoxy/BaTiO_3_ [29], PVA/NBC [26], epoxy/Al_2_O_3_ [44], or HNBR/CB [45]. Moreover, an interphase width of 30 ± 2 nm and 42 ± 3 nm obtained for untreated and treated NPs respectively agrees with previous studies reporting an interphase width of 25 nm for epoxy/BaTiO_3_ [29], between 13 and 16 nm for PVA/NBC [26] and 19 ± 8 nm for HNBR/CB [45].

**Figure 5:**
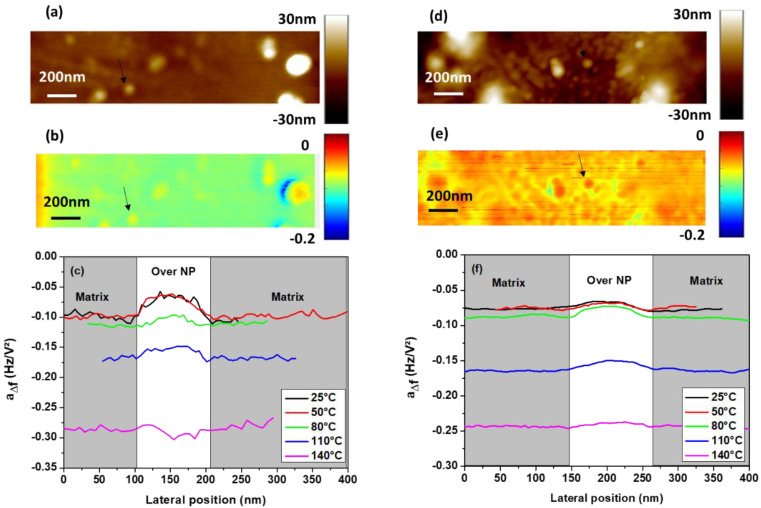
(**a**) Topography, (**b**) frequency shift parameter a_Δf_ at 25 °C and (**c**) frequency shift parameter *a_Δf_* profiles at various temperatures for untreated Si_3_N_4_ nanoparticles. (**d**) Topography, (**e**) frequency shift parameter *a_Δf_* at 25 °C and (**f**) frequency shift parameter *a_Δf_* profiles at various temperatures for treated Si_3_N_4_ nanoparticles.

Figure 5 compares surface topography (Figure 5a,d) and frequency shift parameter *a*_Δ*f*_ (Figure 5b,e) maps acquired for both treated and untreated PI/Si_3_N_4_ NCs in EFM mode at 25 °C. Arrows are used to identify isolated NPs. For both treated and untreated NPs, the presence of an embedded NP induces a higher frequency shift parameter *a*_Δ*f*_ compared to the matrix one. This implies that the apparent dielectric permittivity over NPs is lower than in the PI bulk. As the NP’s dielectric permittivity is higher than that of PI (Table 1), the lower permittivity value is attributed to the interphase. At room temperature, an interphase with dielectric permittivity lower than in the matrix was already observed for other NC materials [28].

Concerning NC with untreated NPs, the temperature dependence of the frequency shift profile over an isolated NP is depicted in Figure 5c. The same profile is observed for 25 and 50 °C, which shows that there is no real modification of the relative dielectric permittivity of both the interphase and the matrix at this temperature. For temperatures higher than 50 °C, the frequency shift parameter baseline, which corresponds to the matrix, decreases with temperature. This implies that the dielectric permittivity of the matrix increases with temperature. Moreover, the bump amplitude on the profile, attributed to the combined effect of the interphase and NPs, tends to disappear when the temperature increases. At 140 °C, the NP/interphase influence is no longer observable. The same behavior is observed for NC with treated NPs (Figure 5f). However, at 140 °C the NP/interphase influence is yet a little bit visible. Indeed, the hypothesis of a retarding effect of the silane treatment compared to untreated NPs is addressed.

**Figure 6:**
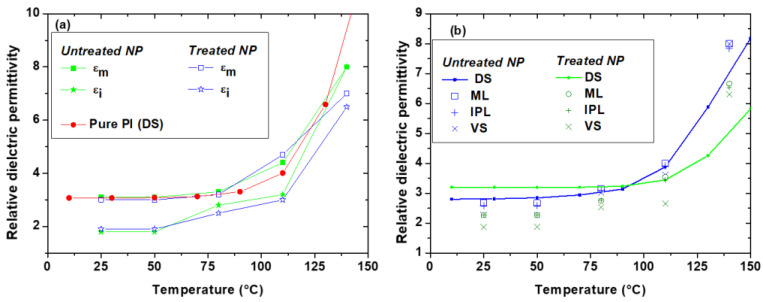
(**a**) Evolution of the relative dielectric permittivity of the matrix (*ε_m_*) and interphase (*ε_i_*) as function of temperature. Comparison is done with pure PI permittivity obtained by DS (0.1 Hz). (**b**) Comparison of the evolution of the experimental (DS at 0.1 Hz on untreated and treated NPs) and the computed apparent relative dielectric permittivity, with various models, as a function of temperature.

To go further, the static dielectric permittivity of the matrix and the interphase are extracted from frequency shift parameter *a*_Δ*f*_ profiles (Figure 5b,e) using the 2D-axisymetric FEM model combined with Equations (3)–(7). Concerning the PI matrix, results obtained from local measurements (EFM) are plotted in Figure 6a and compared to the dielectric permittivity of PI obtained from macroscopic DS measurements. It can be noticed that the trends in temperature for the nanoscale data ($\varepsilon_{m}$) are similar for both treated and untreated NCs and are in good agreement with the PI macroscopic permittivity evolution.

For the determination of $\varepsilon_{i}$, the interphase width *W_i_* is fixed at 30 and 40 nm for untreated and treated NPs, respectively, according to PF-QNM results. As a first approximation, it is considered that *W_i_* is not modified with the temperature, as the temperature remains lower than the glass transition temperature ranges of PI (T_g_ > 370 °C) [41]. Using these width values, the temperature dependence of the interphase permittivity is reported on Figure 6a. At room temperature, the dielectric permittivity of the interphase is lower than the matrix one. This behavior was extensively predicted in previous studies at macroscale by combining modeling and dielectric spectroscopy for various NCs as epoxy/Al_2_O_3_ [22] or LDPE/Al_2_O_3_ [24], and more rarely at nanoscale using EFM as for PE/TiO_2_ [28]. Within the interphase area, the lower dielectric permittivity combined with the higher Young’s modulus compared to the matrix indicates that polymer chains present a better organization at the interphase [46] and/or a restricted movement due to the interaction with NPs [47]. Moreover, for both NC, $\varepsilon_{i}$ increases with temperature. For the untreated NPs, $\varepsilon_{i}\;$reaches the same value as PI at 150 °C, whereas it remains a little bit lower for treated NPs. At around 150 °C, the interphase, the matrix and the NPs have quite the same relative dielectric permittivity, which could explain the fact that all materials have same breakdown strength at 200 °C (Figure 3b).

### 3.3. Impact of Interphase Properties on NC Dielectric Performances

As reported in the literature, classical effective medium theories have failed to predict the NC apparent dielectric permittivity either because only the matrix and the filler are considered [48,49,50,51] or because the interphase dimension and/or dielectric permittivity is unknown [52,53]. Based on the new data here produced, the NC apparent dielectric permittivity is investigated by combining nano- and macro-scale approaches. Indeed, the NC apparent dielectric permittivity $\varepsilon_{r}$ should be computed using those of the matrix, the NPs, and the interphase. This determination is not straightforward and various models, considering the interphase, have been developed for NCs [52,53,54,55,56,57]. Three models are selected and described in the following.

- The simple mixing law (ML) model based on well-known volume fraction average [54]:

(8) $$\varepsilon_{r}=f_{m}\varepsilon_{m}+f_{NP}\varepsilon_{NP}+f_{i}\varepsilon_{i},$$

where *f_m_*, *f_NP_*, and *f_i_* are the volume fraction of the matrix, the NPs, and the interphase respectively.

The volume fractions, reported in Table 2, are determined using the interphase width and the NP weight fraction. As the NP weight fraction is the same for treated and untreated NPs (i.e., 1 wt.%), *f_NP_* is constant. For treated NPs, the interphase is thicker than for untreated NPs, which implies that the interphase volume fraction *f_i_* is higher.

- The Interphase Power Law (IPL) model considers the NP shape [52]:

(9) $$\varepsilon_{r}^{\beta }=f_{m}\varepsilon_{m}^{\beta }+f_{NP}\varepsilon_{NP}^{\beta }+f_{i}\varepsilon_{i}^{\beta },\;$$

where β=1/3 for spherical NPs.

The volume fractions are the same as for the ML model.

- The Vo and Shi model (VS) [53] is based on the theory of polarizability in dielectric materials. This more complex model demonstrates that the NC apparent dielectric permittivity is driven not only by the dielectric permittivity of each component (i.e., the NP and the matrix) but also by the degree of interaction between the NP and the matrix (i.e., the interphase). This interaction is quantified using the parameter *K,* which is expressed in Equation (10):

(10) $$K=\frac{f_{i}}{f_{NP}f_{m}}\;,\;$$

The parameter *K* values are computed and reported in Table 2 for both treated and untreated NPs. This parameter is higher for treated NPs, reflecting a higher degree of interaction between NPs and the matrix when the NP functionalization is done.

In this model, the apparent dielectric permittivity is defined as the following [53]:

(11) $$\varepsilon_{r}=\frac{h+2l}{h-l}\;,\;$$

where *h* and *l* are parameters defined by Equations (12) and (13), respectively:

(12) $$h=1+2\frac{\left(\varepsilon_{m}-\varepsilon_{i}\right)\left(\varepsilon_{i}-\varepsilon_{NP}\right)}{\left(2\varepsilon_{m}+\varepsilon_{i}\right)\left(2\varepsilon_{i}+\varepsilon_{NP}\right)}A-2\frac{\left(\varepsilon_{m}-1\right)\left(\varepsilon_{m}-\varepsilon_{i}\right)}{\left(\varepsilon_{m}+2\right)\left(2\varepsilon_{m}+\varepsilon_{i}\right)}B\;-2\frac{\left(\varepsilon_{m}-1\right)\left(\varepsilon_{m}+2\varepsilon_{i}\right)\left(\varepsilon_{i}-\varepsilon_{NP}\right)}{\left(\varepsilon_{m}+2\right)\left(2\varepsilon_{m}+\varepsilon_{i}\right)\left(2\varepsilon_{i}+\varepsilon_{NP}\right)}f_{NP}\;,\;$$

(13) $$l=\frac{\varepsilon_{m}-1}{\varepsilon_{m}+2}\left(1+2\frac{\left(\varepsilon_{m}-\varepsilon_{i}\right)\left(\varepsilon_{i}-\varepsilon_{NP}\right)}{\left(2\varepsilon_{m}+\varepsilon_{i}\right)\left(2\varepsilon_{i}+\varepsilon_{NP}\right)}A\right)\;-\frac{2\varepsilon_{m}+1}{\left(\varepsilon_{m}+2\right)\left(\varepsilon_{m}+\varepsilon_{NP}\right)}\;\left[\left(\varepsilon_{m}-\varepsilon_{i}\right)+\frac{\left(\varepsilon_{m}+2\varepsilon_{i}\right)\left(\varepsilon_{i}-\varepsilon_{NP}\right)}{2\varepsilon_{i}+\varepsilon_{NP}}A\right]B\;,\;$$

with $A=\frac{1+Kf_{NP}}{1+K}$ and $B=\left(1+K\frac{1-f_{NP}}{1+Kf_{NP}}\right)f_{NP}.$

Equations (8), (9) and (11)–(13) are used to compute the NC apparent dielectric permittivity. $\varepsilon_{m}$ and $\varepsilon_{i}$ are the matrix and interphase permittivities obtained from EFM measurements at different temperatures while $\varepsilon_{NP}$, the inorganic NP permittivity, is supposed to remain constant ($\varepsilon_{NP}\;$= 7.5) in the studied temperature range. Figure 6b compares the temperature dependence of the NC apparent relative dielectric permittivity computed by different laws and measured by DS. Concerning NC with untreated NP, a good agreement is obtained between DS measurements and both ML and IPL models with a slightly better fit for the IPL model. However, the VS model underestimates the apparent dielectric permittivity. By using the PI permittivity determined by DS and by applying the IPL model in the high temperature range, we determine that the interphase permittivity remains constant between 150 and 200 °C (results not shown). Concerning NC with treated NP, all models underestimate the apparent dielectric permittivity compared to the DS measurements and this observation is more visible for the VS model. Different hypotheses could explain this result: (i) the interphase thickness is not constant with temperature when NP are treated with the coupling agent, (ii) a more complex model is needed for the interphase description (two layers’ model for example), and/or (iii) the silane coupling agent has an influence on the matrix properties.

Finally, the relationship between the breakdown field results and the apparent dielectric permittivity is not straightforward. Indeed, at the macroscale, a breakdown strength improvement could be concomitant with the apparent dielectric permittivity increase or decrease [55].

- At low temperature (25 °C < T < 150 °C): $\varepsilon_{i}<\varepsilon_{m}<\varepsilon_{NP}$. This implies that, under an applied bias, the electric field is enhanced in the interphase area where the space charge accumulation is improved. This could be related to the observed breakdown field lowering in NC and explain why it is more pronounced for treated NP where the interphase volume is larger.
- At T = 200 °C: $\varepsilon_{i}\approx \;\varepsilon_{m}\;\approx \varepsilon_{NP}$. Here, under an applied bias, the electric field is homogeneously distributed in the entire NC film. As a consequence, the PI and NC films exhibit the same dielectric breakdown.
- At high temperature (T > 200 °C): $\varepsilon_{m}>\varepsilon_{NP}$. The interphase dielectric permittivity remains unknown (as EFM measurement is no more possible). Moreover, the dielectric breakdown is improved for all NCs compared to neat PI and under an applied bias, the electric field distribution should be higher in NPs than in the matrix ($\varepsilon_{m}>\varepsilon_{NP}$). This configuration seems appropriate.

## 4. Conclusions

In this paper, a multiscale characterization probes the dielectric properties of interphase at various temperatures and evaluates its impact on NC behavior. Macroscale results show that when the temperature increases, PI/Si_3_N_4_ NC exhibits a lower apparent dielectric permittivity and a higher breakdown strength than PI. More particularly, at 300 °C the breakdown strength is improved by a factor of 2 compared to neat PI. From the nanoscale characterization, the interphase thickness is evaluated to 30 ± 2 nm and 42 ± 3 nm for untreated and treated NP, respectively. Moreover, the interphase dielectric permittivity is lower than the matrix one and increases with temperature to reach the same value as the PI matrix at 150 °C.

The comparison of macro- and nano-scale measurements emphasizes that in order to accurately reproduce the NC apparent dielectric permittivity, the IPL model should be preferred mainly for untreated NPs.

Finally, a strong correlation is observed between the breakdown strength and the interphase dielectric permittivity evolution with temperature. Consequently, the obtained results provide new insights into the influence of the interphase properties on the NC dielectric properties at the macroscale, mainly at high temperature.

## Figures and Tables

**Table 1:** Materials properties: density, NP radius, and relative static dielectric permittivity *ε_r_* (i.e., dielectric permittivity at low frequency).

	Density (g∙cm^−3^)	NP Radius (nm)	*ε_r_*
PI	1.48	/	3
Si_3_N_4_	2.67	10–20	7.5

**Table 2:** The matrix (*f_m_*), NPs (*f_NP_*), and interphase (*f_i_*) volume fractions as well as the interaction parameter *K* for untreated and treated nanocomposites.

	*f_m_*	*f_NP_*	*f_i_*	*K*
Untreated NPs	0.644	0.005	0.351	109
Treated NPs	0.306	0.005	0.689	451

## Data Availability

The data that support the findings of this study are available from the corresponding author upon reasonable request.
